# Branched microtubule nucleation and dynein transport organize RanGTP asters in *Xenopus laevis* egg extract

**DOI:** 10.1091/mbc.E23-10-0407

**Published:** 2023-11-22

**Authors:** Jacopo Scrofani, Felix Ruhnow, Wei-Xiang Chew, Davide Normanno, Francois Nedelec, Thomas Surrey, Isabelle Vernos

**Affiliations:** a Quantitative Cell Biology Program, Centre for Genomic Regulation (CRG), The Barcelona Institute of Science and Technology, 08003 Barcelona, Spain; b Sainsbury Laboratory, Cambridge University, Bateman street, CB2 1LR Cambridge, UK; c Universitat Pompeu Fabra (UPF), 08002 Barcelona, Spain; d Institución Catalana de Investigación y Estudios Avanzados (ICREA), Pg. Lluis Companys 23, 08010 Barcelona, Spain; Indiana University

## Abstract

Chromosome segregation relies on the correct assembly of a bipolar spindle. Spindle pole self-organization requires dynein-dependent microtubule (MT) transport along other MTs. However, during M-phase RanGTP triggers MT nucleation and branching generating polarized arrays with nonastral organization in which MT minus ends are linked to the sides of other MTs. This raises the question of how branched-MT nucleation and dynein-mediated transport cooperate to organize the spindle poles. Here, we used RanGTP-dependent MT aster formation in *Xenopus laevis* (*X. laevis*) egg extract to study the interplay between these two seemingly conflicting organizing principles. Using temporally controlled perturbations of MT nucleation and dynein activity, we found that branched MTs are not static but instead dynamically redistribute over time as poles self-organize. Our experimental data together with computer simulations suggest a model where dynein together with dynactin and NuMA directly pulls and move branched MT minus ends toward other MT minus ends.

## Abstract (executive summary)

Spindle pole assembly requires the dynein-mediated transport of microtubules (MTs). Branching inside the spindle generates MTs linked to large protein complexes on their minus ends. Therefore, how dynein organizes branched MTs remains unknown.

We combined RanGTP-dependent MT aster formation in *X. laevis* egg extract with advanced imaging techniques and computer simulations and found that branched MTs are organized dynamically by dynein to form the pole of an aster.

These results suggests a novel mechanism where dynein together with dynactin and NuMA directly pulls and move branched MT minus ends toward other MT minus ends.

## INTRODUCTION

During cell division, a bipolar spindle assembles to segregate the genetic material. An important organizing principle in spindle assembly is the focusing of microtubule (MT) minus ends to form the two poles. In most somatic animal cells centrosomes organize the spindle poles by providing a focus of nucleating activity from which MTs radiate. Centrosomes are however not essential for bipolar spindle assembly and pole focusing, even in somatic cells (Heald *et al.*, 1996; Khodjakov *et al.*, 2000; Karsenti and Vernos, 2001; Megraw *et al.*, 2001; Mahoney *et al.*, 2006; Wong *et al.*, 2015). In mitotic and meiotic cells, MT minus end pole focusing requires dynein, a MT minus end – directed motor, the multisubunit dynactin complex and the potential adaptor protein NuMA as shown in different cell types and in *Xenopus* egg extract (Merdes *et al.*, 1996; Mitchison *et al.*, 2005; Raaijmakers and Medema, 2014; Hueschen *et al.*, 2017; Reck-Peterson *et al.*, 2018; So *et al.*, 2022).

Dynein is thought to concentrate MT minus ends into poles via a motor mediated self-organization process (Merdes *et al.*, 1996; Merdes *et al.*, 2000; Mitchison *et al.*, 2005). This notion is supported by the observation that randomly oriented MTs that are artificially nucleated by taxol or DMSO in *X. laevis* egg extract assemble into asters where the minus ends are clustered together in a strictly dynein-dependent manner (Verde *et al.*, 1991; Wilde and Zheng, 1999). In vitro reconstitutions with purified proteins further demonstrated that MT crosslinking motors were sufficient to dynamically organize MTs into asters (Nedelec *et al.*, 1997; Surrey *et al.*, 2001). However, spindle assembly does not rely on random MT nucleation but on a nucleation pathway activated by RanGTP around chromosomes (Meunier and Vernos, 2016), a process involving factors including TPX2, Aurora-A, NEDD1, RHAMM, the multisubunit Augmin complex and the γ-Tubulin Ring Complex (γ-TuRC; Wittmann *et al.*, 2000; Petry *et al.*, 2013; Scrofani *et al.*, 2015; Timon Perez *et al.*, 2022).

RanGTP triggers MT branching through the nucleation of MTs from preexisting MTs in a nearly parallel orientation. This nucleation mode leads to large polarized MT arrays with roughly uniform MT orientation, as observed in *X. laevis* egg extract containing RanGTP when dynein is inhibited (Wilde and Zheng, 1999; Clausen and Ribbeck, 2007; Petry *et al.*, 2013; Thawani *et al.*, 2019; Gai *et al.*, 2021). Branched MT nucleation has also been observed in spindles (Decker *et al.*, 2018; Kaye *et al.*, 2018; David *et al.*, 2019; Verma and Maresca, 2019; Almeida *et al.*, 2022). In mitotic HeLa cells Augmin-dependent γ-tubulin nucleation complexes move toward the spindle poles (Lecland and Luders, 2016). In plants Augmin-dependent MT branches become mobile after katanin dependent severing (Yagi *et al.*, 2021). Although there are indications suggesting that MT branches are not static there is a clear gap of our understanding about how the seemingly very different organizational principles of motor-mediated MT self-organization and branched MT nucleation could work together. How dynein organizes branched MTs to form poles remains an open question.

Here, we monitored RanGTP-dependent MT aster formation in *X. laevis* egg extract to study the interplay between dynein and branched MT nucleation during pole self-organization. Using temporally controlled perturbations of MT nucleation and dynein activity in combination with live imaging and computer simulations, we show that dynein dynamically redistributes branched MTs over time as poles self – organize suggesting a novel model for the self-organization of poles under physiological conditions.

## RESULTS

### Inhibition of dynein prevents pole focusing in RanGTP egg extract

**FIGURE 1:**
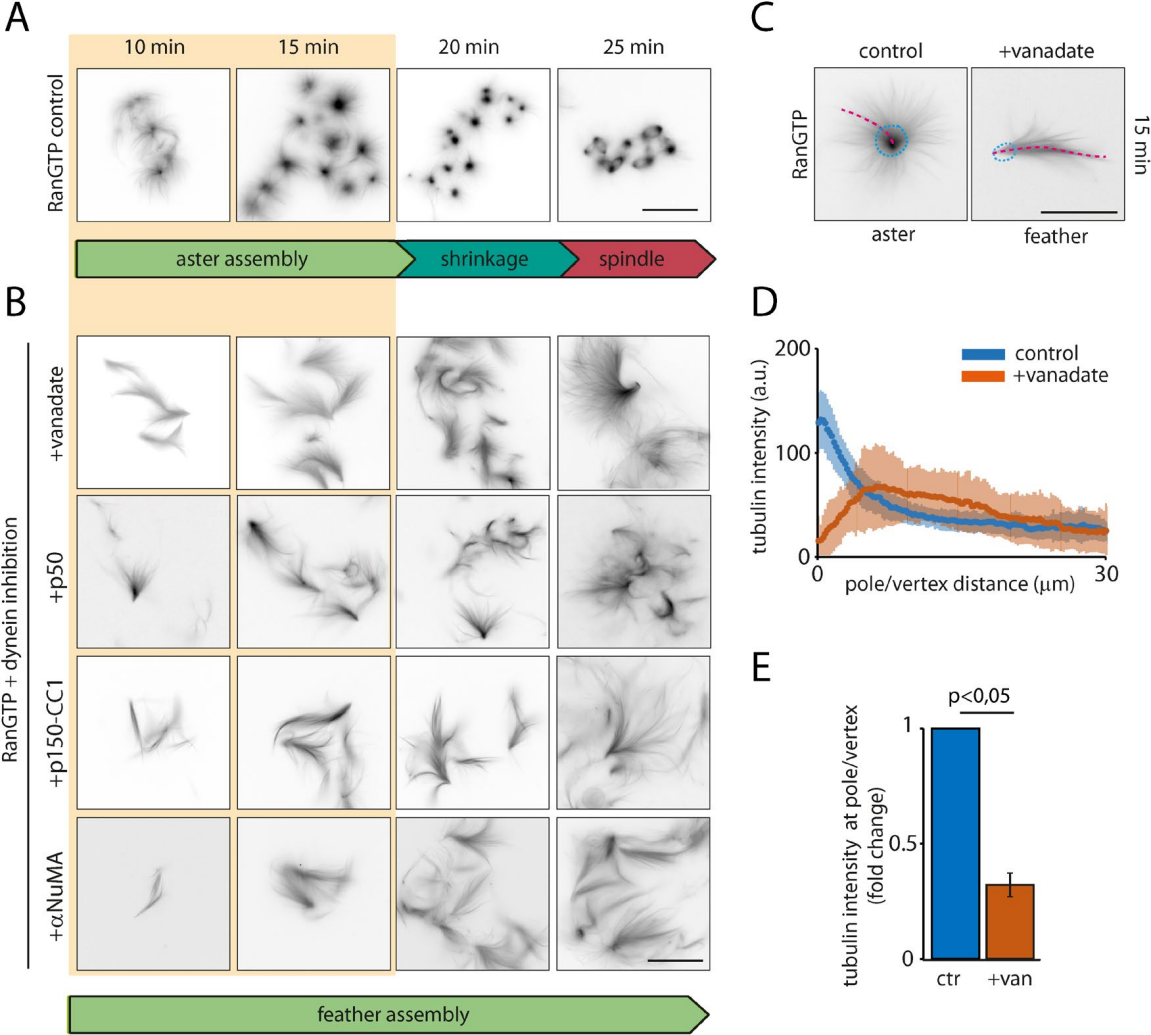
Self-organization of asters in the *X. laevis* egg extract**.** (A) MT structures forming in egg extracts incubated with RanGTP (control) or (B) in the presence of different dynein inhibitors as indicated. Samples were collected and fixed at the indicated time points. The different phases of MT self-organization are indicated at the bottom of each panel. Shaded area highlight the aster self-organization phases. MTs were visualized through rhodamine-tubulin fluorescence. Scale bar, 50 μm. (C) Representative RanGTP aster (control) and feather (+vanadate). Dashed lines and circles indicate the selected area used for quantifications. MTs were visualized through rhodamine-tubulin fluorescence. Scale bar, 50 μm. (D) Tubulin intensity distribution on RanGTP asters and feathers from radial selections as shown in (A). The intensity profiles were obtained from radial selection as indicated by red dotted lines. Data points correspond to average values and shaded areas to SD from 60 (control) and 55 (+vanadate) profiles from one representative experiment. (E) Tubulin intensity on asters poles and feathers vertexes measured on circular selections as shown in (A). The bar graph represent the fold change over the control of the average intensity measured in three independent experiments. Error bar is SD. *p*: Student’s *t* test. *n* = 340 asters and 312 feathers.

To investigate the mechanism that drives spindle pole organization, we studied MT aster formation in meiotic M-phase *X. laevis* egg extract as a model system. As previously observed (Verde *et al.*, 1991; Wilde and Zheng, 1999), MTs nucleated by adding Paclitaxel or DMSO to the egg extract formed asters with focused poles (Supplemental Figure S1, A and B). Dynein inhibition by adding vanadate generated disorganized MT structures, confirming that dynein activity is required for the organization of randomly nucleated MTs into asters (Supplemental Figure S1, A and B; Schroer *et al.*, 1989; Verde *et al.*, 1991; Wilde and Zheng, 1999). Addition of constitutively active RanGTP to the M-phase egg extract also promoted the self-organization of MTs into asters (Figure 1A). However, and in contrast to the taxol or DMSO induced asters, RanGTP asters were not stable over time. Imaging the MT structures fixed at different times after RanGTP addition or imaging dynamically evolving structures mounted between passivated glass surfaces showed that asters shrank in size after around 15 min and rearranged into mini spindle-like structures after around 25 min (Figure 1A; Movie 1; Wilde *et al.*, 2001; Rosas-Salvans *et al.*, 2018). Dynein inhibition by addition of vanadate to the reaction interfered with aster organization, leading to the formation of elongated “feather”-like structures as a consequence of branched MT nucleation (Figure 1B; Movie 1), as reported previously (Wilde and Zheng, 1999; Petry *et al.*, 2013; Gai *et al.*, 2021). MT feathers did not contain any pole-like structure (Figure 1C), as indicated by a low tubulin density at the vertex of the feathers in contrast to the high density measured at the center of asters (Figure 1, D and E). MT feathers grew in size over time, but did not evolve into mini-spindles (Figure 1B; Movie 1). Asters and feathers are, therefore, morphologically and functionally different. Moreover, feathers consisting of branched MTs only formed when dynein was inhibited and therefore did not appear to be normal intermediates of assembling RanGTP asters (Figure 1, A and B; Movie 1).

**Figure:** Movie S1 **RanGTP asters and feathers assembly** Live confocal fluorescence microscopy of RanGTP aster (left) and feather (right) assembly. Samples were squashed between PLL‐passivated glass. MTs were visualized through Atto647‐tubulin fluorescence. Images were collected every 15 s. Time frame 15 fps. Scale bar, 50 μm.

MT feathers were also obtained by interfering with dynein activity by adding other established inhibitors of dynein to the extract, such as recombinant p50/dynamitin, the CC1 fragment of p150 (both components of the dynactin complex) or an anti-NuMA tail anti­body serum (Figure 1B; Merdes *et al.*, 1996; Wittmann and Hyman, 1999; King *et al.*, 2003). This demonstrates that the “feather” phenotype is specific for dynein inhibition in RanGTP extract and raises the question of how dynein activity and MT branching cooperate under normal conditions to organize a pole.

### NEDD1 concentrates rapidly at the forming poles during RanGTP aster self-organization

**FIGURE 2:**
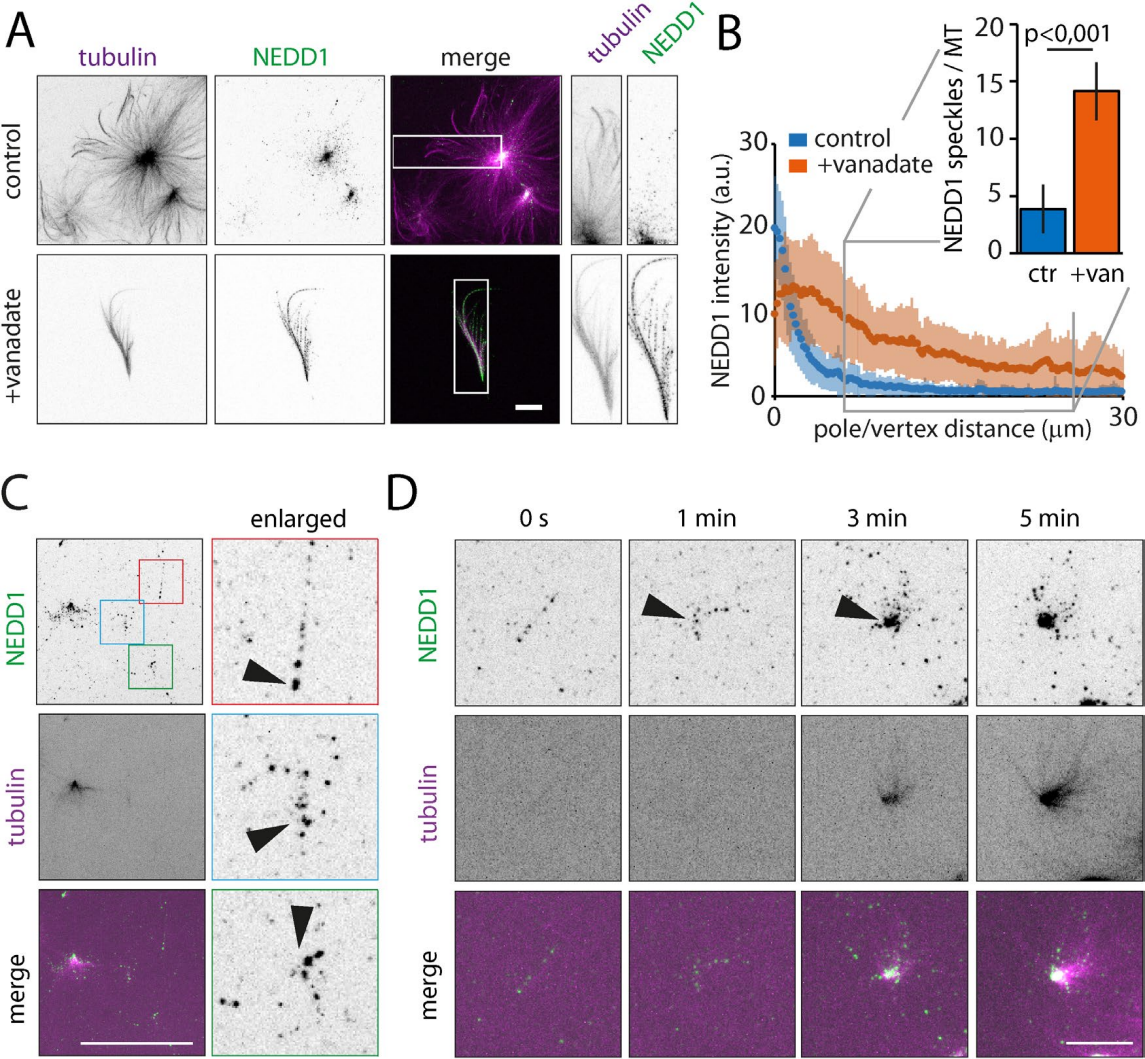
NEDD1 localization revealed early RanGTP aster self-organization. (A) NEDD1 localization on RanGTP asters (control) and feathers (+vanadate) in fixed samples. NEDD1 is visualized with an anti-NEDD1-Atto488 antibody that was added to the extract (green). MTs were visualized through rhodamine-tubulin fluorescence (magenta). Scale bar, 10 μm. (B) NEDD1 distribution profile on asters (control) and feathers (+vanadate). The profiles were obtained from line selections on the pole/vertex to periphery axis. Data points are the average intensity and shaded areas standard deviations from *n* = 50 (control) and *n* = 50 (+vanadate) profiles in one representative experiment. The bar plot (inset) shows the average number of NEDD1 speckles counted on 20-μm long MT segments (grey selection) in control asters (ctr) and vanadate feathers (+van). Error bars indicate SD. *n* = 63 (control) and *n* = 55(+vanadate) MTs from three independent experiments were analyzed. *p*: Student’s *t* test. (C) Selected examples of NEDD1 on early RanGTP aster intermediates. Images are maximum intensity projections. Red, blue, and green squares highlights enlarged regions (right). Arrows indicate the position of the new forming poles. MTs were visualized through Atto647-tubulin fluorescence (magenta) and NEDD1 via the anti-NEDD1-Atto488 antibody (green). Scale bar, 20 μm. (D) Live confocal microscopy of early RanGTP aster self-organization. Images are maximum intensity projections at the indicated times. Arrows indicate the position of the new forming pole. MTs were visualized through Atto647-tubulin fluorescence (magenta in composite images) and NEDD1 via the anti-NEDD1-Atto488 antibody (green in composite images). Scale bar, 20 μm.

NEDD1 is an essential component of the MT nucleation complex that binds to the lattice of a preexisting MT targets and activates branched MT nucleation in a RanGTP dependent manner (Luders *et al.*, 2006; Pinyol *et al.*, 2013; Scrofani *et al.*, 2015). To further investigate the mechanism by which dynein contributes to branched MTs organizing into a pole we used a fluorescently labeled polyclonal antibody against *X. laevis* NEDD1 that does not interfere with RanGTP aster and mini-spindle assembly (Supplemental Figure S2, A and B; Scrofani *et al.*, 2015). Bright NEDD1 speckles predominantly accumulated at the center of RanGTP asters while very few speckles were scattered along the MTs (Figure 2, A and B). In contrast, the NEDD1 speckles were rather evenly distributed all along the MTs in RanGTP feathers (Figure 2, A and B). Consistently, TPX2 and Aurora A which are part of the same RanGTP-dependent nucleation complex as NEDD1 (Scrofani *et al.*, 2015) showed similar distributions on asters and feathers (Supplemental Figure S2, C–F). These results indicate that dynein activity is essential for the accumulation of the branched-MT nucleation complexes toward the poles.

We then used live imaging to follow the dynamics of NEDD1 localization in RanGTP extract squashed between passivated glasses using the fluorescently labeled anti-NEDD1 antibody. The bright fluorescent NEDD1 speckles allowed the observation of early RanGTP aster intermediates before MTs became visible. RanGTP asters assembled from small clusters of NEDD1 suggesting structures of very few MTs (Figure 2C). NEDD1 speckles were scattered along MTs and accumulated within a few minutes at the position of the newly forming pole (Figure 2D). This observation suggests that, when dynein is active, branched-MT nucleation complexes are transported toward MT minus ends therefore defining the position of a new pole.

### NEDD1 moves toward RanGTP aster poles

**FIGURE 3:**
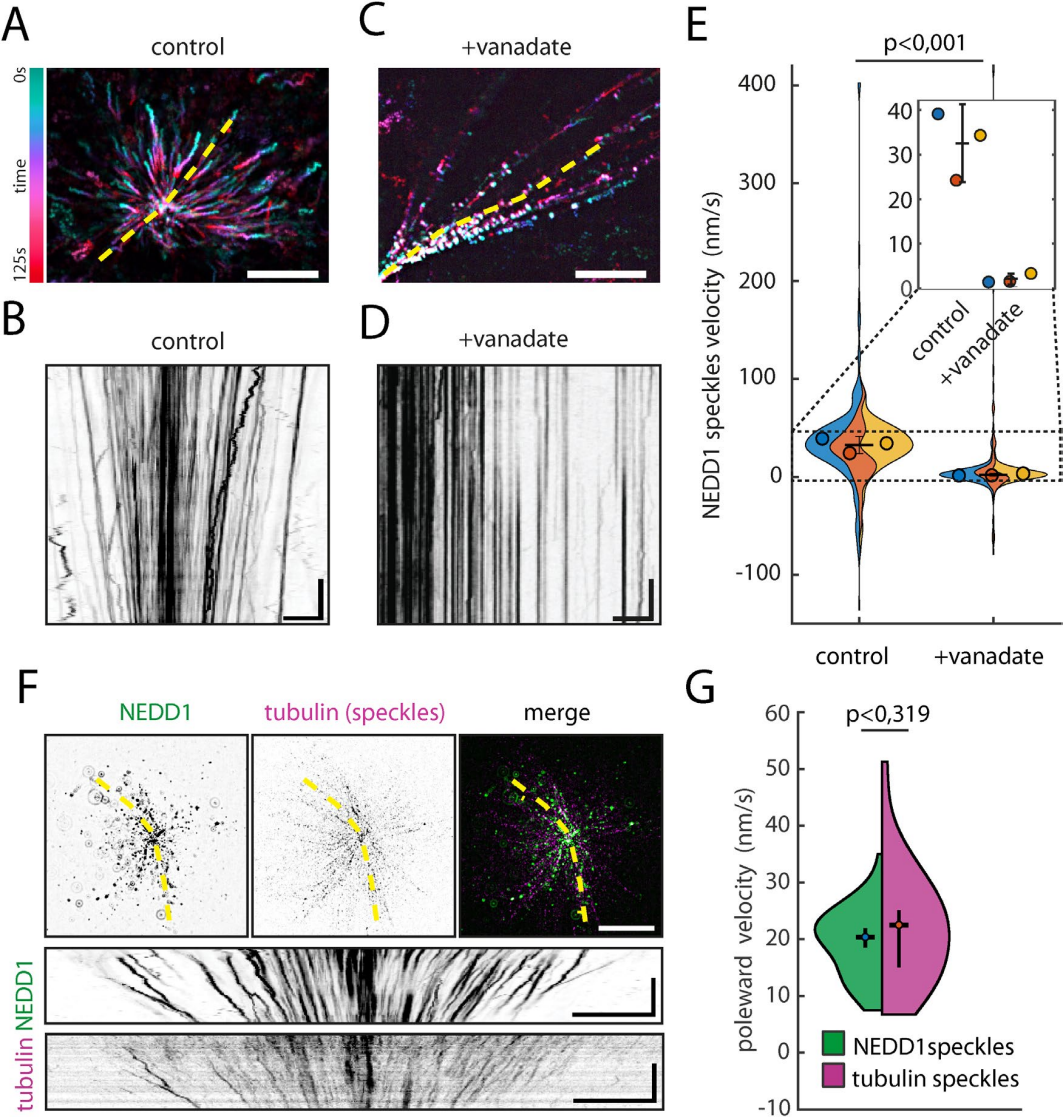
NEDD1 movement on RanGTP asters and feathers. (A and C) Color coded maximum intensity projections from epifluorescence live imaging of NEDD1 speckles on RanGTP asters and feathers. Scale bars: 10 μm. (B and D) Representative kymographs of NEDD1 speckles prepared on yellow dashed line selections as shown in maximum projected images. NEDD1 was visualized via anti-NEDD1-Atto488 antibody. Scale bars: x = 20 s, y = 5 μm. (E) NEDD1 speckle velocity quantification by single particle tracking on RanGTP asters (control) and feathers (+vanadate). Superviolin plot represents probability density of individual speckle velocities. Dots are median values from three asters and feathers imaged in three independent experiments (blue, red, and yellow). Inset is a zoom vison of the dashed area showing the median values. Error bars are the mean of median velocities ± (2*standard error of mean-95% confidence). *p*: Mann-Whitney’s U-test using median velocities from each track. Asters: *N* = 3, *n* = 217 tracks. Feathers: *N* = 3, *n* = 192 tracks. (F) Live epifluorescence microscopy of NEDD1 and MT speckles on RanGTP asters (top). Representative kymographs of NEDD1 and MT speckles from the indicated selection (yellow dashed lines) (bottom). Scale bars: 20 μm (images), x = 120 s, y = 10 μm (kymographs). (G) Speckle velocity measured by PIV on asters. Quantifications correspond to 10 s. Half violin plot represent probability density for the median speeds of NEDD1 and MT speckles (NEDD1: *n* = 18434; tubulin: *n* = 3152) measured in every analyzed aster (NEDD1: *N* = 45; tubulin: *N* = 18) from three independent experiments. Error bars are the mean of median velocities ± 95% confidence. *p*: Mann-Whitney’s U-test.

To visualize directly how NEDD1 accumulates at the poles we pre-assembled RanGTP asters in bulk egg extract, squashed them between passivated coverslips and immediately imaged individual structures. Most NEDD1 speckles moved slowly and persistently toward the aster pole (Figure 3A), as can also be appreciated in a kymograph (Figure 3B), in agreement with minus end directed dynein-driven movement. In contrast, speckles were mainly static on feathers (Figure 3, C and D). We then quantified the instantaneous velocities of the bright NEDD1 speckles toward the pole/vertex by automatic tracking (Supplemental Movie 2). With the exception of those positioned at the very dense aster poles and feather axes, we could track a large number of speckles, which were representative of NEDD1 movement in distal regions of both structures. NEDD1 speckles moved in asters with 32.6 nm/s on average (Figure 3E). Although this is considerably slower than the unloaded dynein speed (McKenney *et al.*, 2014; Schlager *et al.*, 2014; Jha *et al.*, 2017; Elshenawy *et al.*, 2020; Htet *et al.*, 2020; Pelletier *et al.*, 2020), it is known that dynein can slow down considerably when under load (Belyy *et al.*, 2016), providing a potential explanation for the slow dynein-driven movement of NEDD1 toward the pole. We could also observe a small fraction of speckles diverging from the large slow-moving population with faster movement in both poleward and outward directions suggesting that occasionally other motors and/or different loads may determine some NEDD1 velocities (Supplemental Figure S3A). When dynein was inhibited in feathers, the average NEDD1 speckle velocity was reduced by more than an order of magnitude to 2.1 nm/s (Figure 3E), demonstrating that this is the main motor affecting speckle speeds. Occasionally, fast and short movements were also observed in feathers (Supplemental Figure S3B).

**Figure:** Movie S2 **NEDD1 speckles on RanGTP asters and feathers** Live epifluorescence microscopy of RanGTP asters and feathers. Speckles were tracked automatically and their velocity toward the aster center or feather vertex (blue dot) measured. Inward slow (green) and fast (light blue) and outward (magenta) tracks were visualized. NEDD1 was visualized via anti‐NEDD1‐Atto488 antibody staining of the endogenous protein (right). Images were collected every 0.5 s. Time frame 15 fps. Scale bar, 10 μm.

These results suggest that NEDD1 containing MT nucleation complexes, possibly together with branched MTs, are slowly moved by dynein toward the RanGTP aster poles.

### NEDD1 and MTs move together to RanGTP aster poles

To determine whether NEDD1 moves in association with branched MTs we imaged NEDD1 and tubulin speckles by live microscopy. RanGTP asters were assembled in the presence of the fluorescently labeled NEDD1 antibody and a low concentration of fluorescently labeled tubulin. Strikingly, tubulin speckles moved slowly toward the aster poles producing tracks strikingly similar to those of NEDD1 (Figure 3F; Supplemental Movie 3). In MT feathers assembled in the presence of either vanadate or the p150-CC1 fragment, neither NEDD1 nor tubulin speckles moved much (Supplemental Figure S3, C and D; Supplemental Movie 3). Due to the high density of NEDD1 particles and tubulin speckles the automated particle tracking had problems tracing all trajectories. Therefore, we combined single particle tracking and particle image velocimetry (PIV; Raffel *et al.*, 2018) to quantify the overall poleward movement of NEDD1 and tubulin speckles (Supplemental Figure S4). Importantly, we could quantify a large number of speckles including those in the proximity of the particularly dense pole and found that NEDD1 and tubulin speckle velocities were not significantly different (respectively 20.2 nm/s and 23.2 nm/s on the average of asters; *p* = 0.319; Figure 3G). NEDD1 and tubulin speckles spread over a wide range of velocities when measured on individual asters (Supplemental Figure S5A). Moreover, there was a similar and significant positive correlation between tubulin or NEDD1 speckle movement and their distance from the aster pole indicating that NEDD1 and branched MT complexes slow down as they reach the center of the aster (Supplemental Figure S5B). These results suggest that NEDD1 and branched MTs move together in agreement with live imaging of RanGTP feathers where NEDD1 localizes at branched MT minus ends (Supplemental Figure S6; Supplemental Movie 4).

**Figure:** Movie S3 **NEDD1 and tubulin speckles on RanGTP asters and feathers** Live epifluorescence microscopy of RanGTP asters and feathers. NEDD1 was visualized via anti‐NEDD1‐Atto488 antibody staining of the endogenous protein (right). Tubulin speckles were visualized by a low concentration of rhodamine tubulin. Images were collected every 2 s. Time frame 15 fps. Scale bar, 10 μm.

**Figure:** Movie S4 **NEDD1 localization on RanGTP branched MTs minus ends** TIRF microscopy of RanGTP feathers. Video captions are shown in Fig. S6C. NEDD1 was visualized via anti‐NEDD1‐Atto488 antibody staining of the endogenous protein (right). MTs were visualized by Atto647 tubulin. Images were collected every 2 s. Time frame 15 fps. Scale bar, 10 μm.

### Computer simulations of dynein-mediated transport of nucleation complexes recapitulate RanGTP aster and feather assembly

**FIGURE 4:**
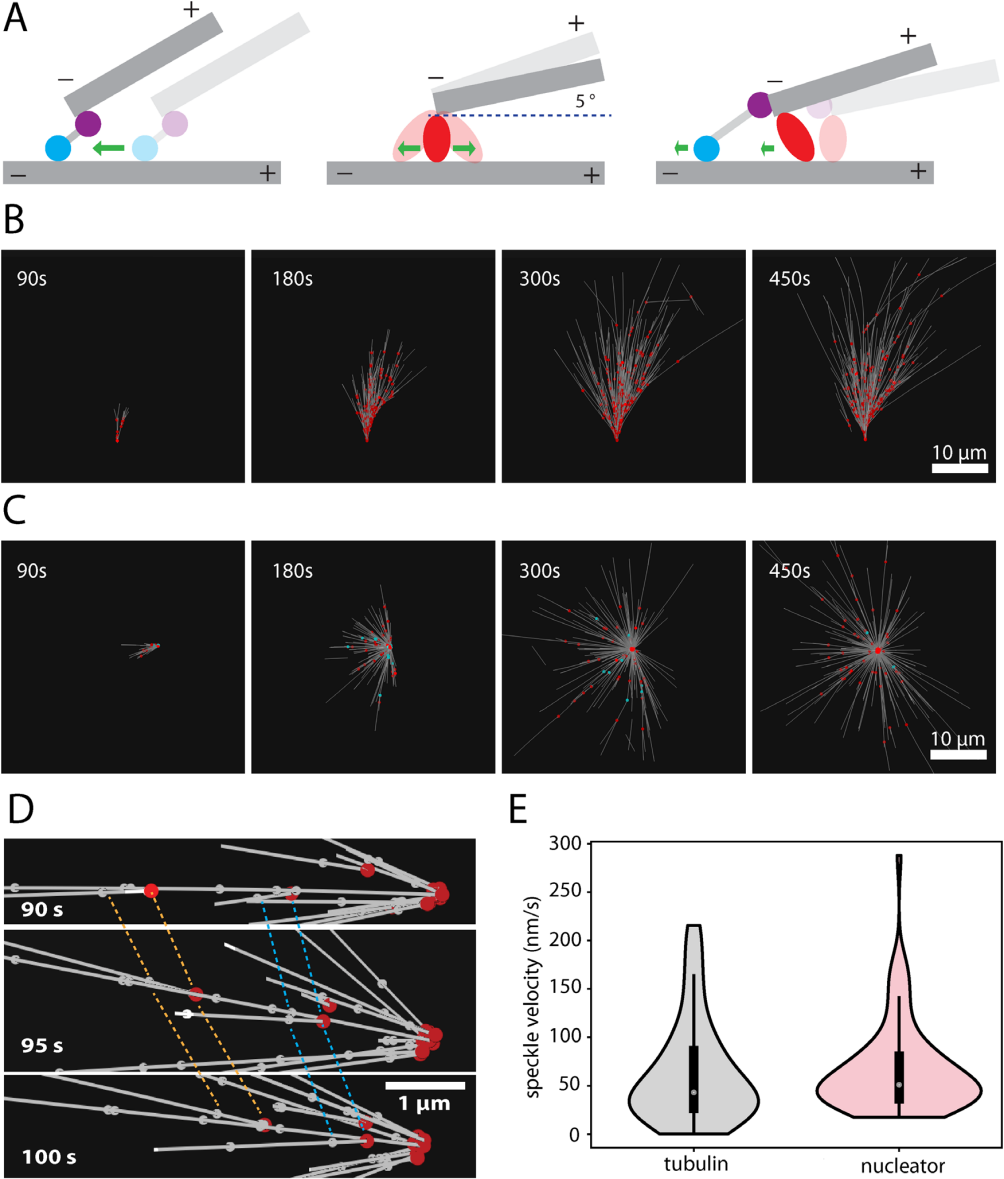
Computer simulation of dynein and branched-MT self-organization. (A) Schematic of the models used in the simulations for minus motors and branching nucleators. Left: a dynein-like motor is composed of a unit (purple) statically bound to a MT minus end, and a minus end-directed motor unit (blue) moving on another MT. Middle: a nucleator (red) generating a new MT at a shallow angle upon contact with an existing MT. The nucleator diffuses along the MT. Right: a dynein-like motor transports a branched MT. (B) Time series of simulated MT feather formation starting from a single initial mother MT in the presence of 270 nucleators (red). (C) Time series of simulated aster formation starting from a single initial mother MT in the presence of 270 nucleators (red) and 5400 dynein-like motors (blue). (D) Close-up view of selected MTs during aster formation as depicted in (C), highlighting the cotransport of MT speckles (white circles) and nucleators (red circles). (E) The speeds of 63 MT speckles and 76 nucleators during the aster formation in (C; measured between 90 and 100 s in one simulation for one aster).

Our results suggest a model where dynein transports NEDD1 and branched MTs toward the pole of an aster. To test this hypothesis more formally, we carried out computer simulations using Cytosim (Francois Nedelec, 2007). The simulations contained three types of components: dynamic MTs, branching MT nucleators, and dynein-like motors (Figure 4A). Branching MT nucleators bind stochastically to preexisting MTs. When bound to a MT, a nucleator initiates the formation of another MT that elongates by plus end growth. The newly nucleated MT remains linked with its minus end to the preexisting MT via the diffusively bound nucleator in a close-to-parallel orientation, forming a “MT branch.” MT plus ends display dynamic instability, that is, they switch stochastically between growth and shrinkage. The simulated dynein is composed of a cargo domain able to stochastically bind to a MT minus end, and a motor domain that can stochastically bind and subsequently move along another MT toward its minus end. Dynein velocity is dependent on force, and is strongly reduced by the friction generated by the MT-bound nucleator at the branch point.

When simulating the branching reaction starting from a single preexisting MT in the presence of nucleators, but in absence of dynein, after several branching events, the system evolved into a feather-like structure within 5 min, very similar to the experimental data in *Xenopus* egg extract in the presence of dynein inhibition (Figure 4B; Supplemental Movie 5). When both dynein motors and nucleators were present in the simulated system, an aster-like structure formed within 8 min (Figure 4C; Supplemental Movie 6). The nucleators became concentrated at the aster pole, very similarly to NEDD1 accumulation in our experiments without dynein inhibition.

**Figure:** Movie S5 **Computer simulation of branched MT nucleation without dynein** MTs (gray) and nucleators (red).

**Figure:** Movie S6 **Computer simulation of branched MT nucleation with dynein** MTs (gray), nucleators (red) and dynein (blue).

We also displayed points randomly placed on the MTs to mimic tubulin speckles in the aster simulations (Figure 4D). The velocity distributions of these speckles and the nucleators as they moved toward the aster pole were almost identical (Figure 4E), and similar to the experimental distributions (Supplemental Figure S5A). This can be understood intuitively given that branching nucleators are also fixed with respect to the MT lattice (the minus end is static), just like the speckles.

Altogether, the simulations recapitulate quite closely the experimental data, supporting a model of dynein-dependent transport of branched MTs and nucleators being an efficient mechanism for aster assembly and pole formation.

### Dynein inhibition blocks NEDD1 and branched MT transport in RanGTP asters

**FIGURE 5:**
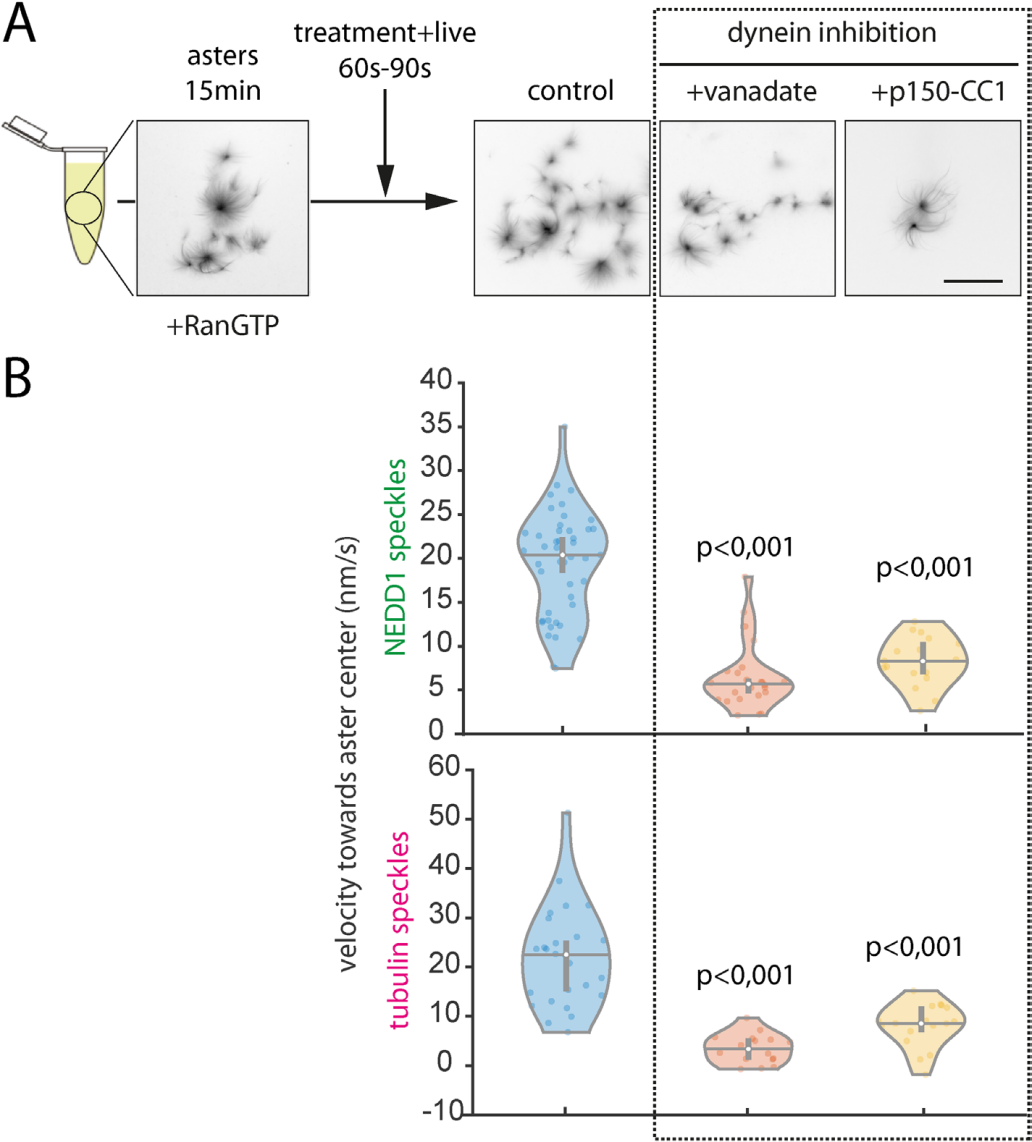
NEDD1 and MT speckles velocity upon dynein inhibition. (A) Experimental set up to measure the effect of dynein inhibition on asters preassembled for 15 min. Representative images from fixed samples taken after live imaging (5 min from aster treatment). MTs were visualized through rhodamine-tubulin fluorescence. Scale bar, 50 μm. (B) Violin plots represent the density probability of NEDD1 (top) and MT (bottom) speckles velocities measured by PIV. Dots are median values for individual asters from three independent experiments. Error bars are the mean of median velocities ± 95% confidence. *p*: Mann-Whitney’s U-test. For NEDD1 speckles: *n* = 45 (control), 24 (vanadate), 17(p150-CC1). For MTs speckles: *n* = 26 (control), 16 (vanadate), 17 (p150-CC1).

To obtain further support for the role of dynein in the transport of branched MTs, we inhibited dynein on RanGTP asters preassembled for 15 min. Dynein inhibition did not disrupt the poles and MTs kept their radial distribution as observed in fixed samples taken 5 min after perturbation (Figure 5A). Dynein inhibition either by vanadate or p150-CC1 reduced dramatically NEDD1 and tubulin speckle velocities measured 1–1.5 min after the addition to nonfixed asters (Figure 5B). Moreover, the correlation of the velocities with pole distance was severely reduced as well (Supplemental Figure S5, B and C). Strikingly, this effect of dynein inhibition on speckle speeds was very fast. These data strongly suggested that dynein is directly involved in the transport of branched MTs on asters.

### Releasing dynein inhibition allows the conversion of MT feathers into asters

**FIGURE 6:**
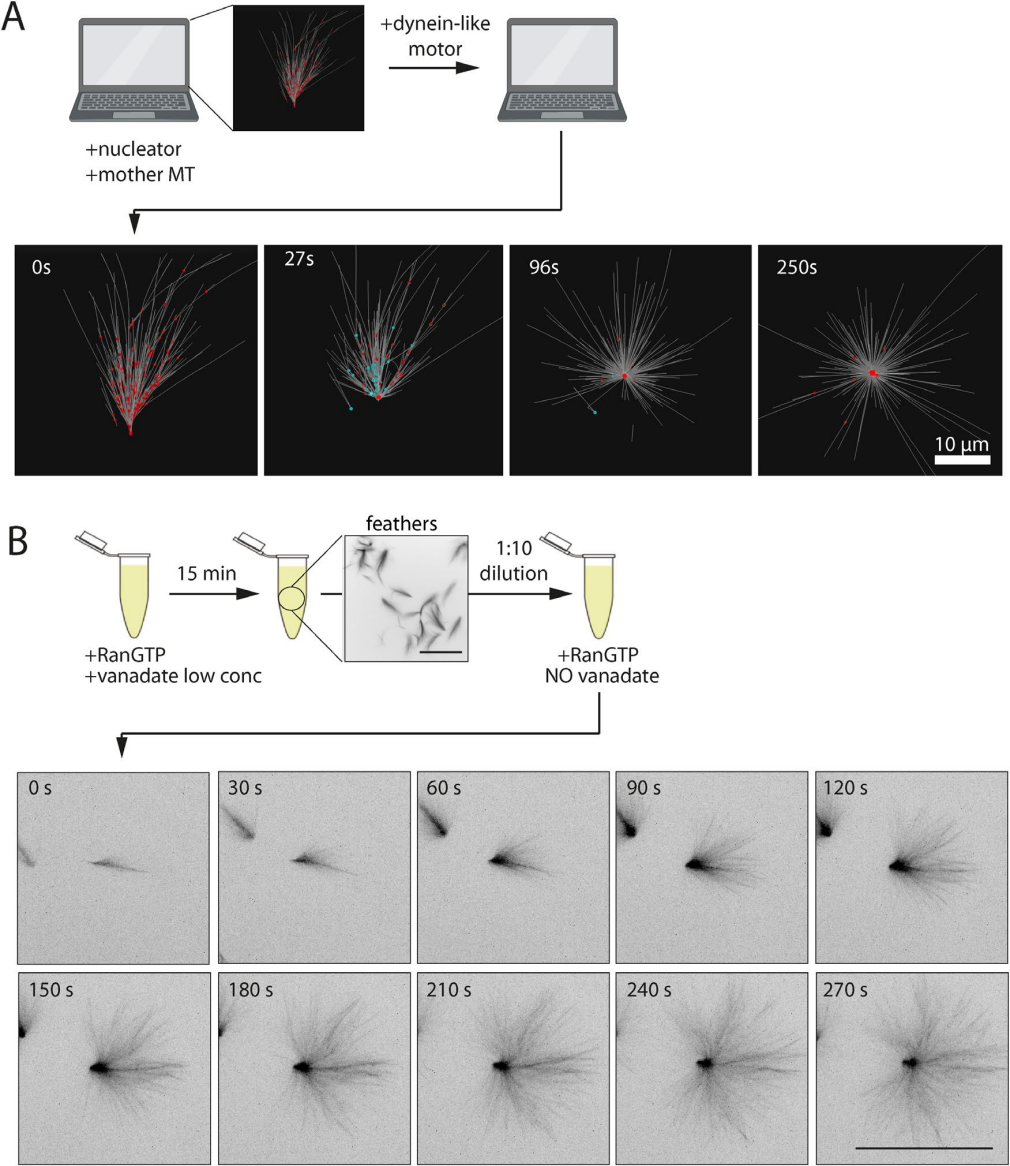
Release of RanGTP feathers from dynein inhibition. (A; Top) Procedure to simulate dynein addback to a feather-like structure. The simulated preformed MT feather is the final assembly of the simulation shown in Figure 4B. (Bottom) Time series of a simulation of the preformed MT feather transforming into an aster after addition of 5400 dynein-like motors to the preformed MT feather. (B; Top) experimental procedure adopted to release RanGTP feathers from dynein inhibition. The image is a representative example of 15 min feathers fixed immediately before the release from dynein inhibition. MTs were visualized through rhodamine-tubulin fluorescence. Scale bar, 50 μm. (Bottom) live confocal fluorescence microscopy imaging of representative RanGTP feathers after release from dynein inhibition (bottom). Images are maximum intensity projections at the indicated time points. MTs were visualized through Atto488-tubulin fluorescence. More than 30 structures were observed in three independent experiments. Scale bar, 50 μm.

Finally, we asked whether dynein could reorganize preformed feathers into asters. We simulated the assembly of a feather and added then dynein to the system. Strikingly, the feather transformed into an aster within 4 min (Figure 6A; Supplemental Movie 7). The branched MTs and nucleators moved toward and accumulated at the forming aster pole, while the angular MT distribution progressively increased.

**Figure:** Movie S7 **Computer simulation of feather MT organization after dynein addition** MTs (gray), nucleators (red) and dynein (blue).

To test this prediction experimentally we first determined the lowest concentration of vanadate required to drive the assembly of feathers (Supplemental Figure S7A). Using this concentration (25 μM vanadate), we induced feather assembly by addition of RanGTP and then released dynein inhibition by dilution with fresh egg extract complemented with RanGTP, thereby decreasing the inhibitor concentration below the threshold for feather assembly. Samples fixed at different times after dilution showed that feathers evolved into asters within a few minutes and then later organized into mini-spindles indicating that the dynein-dependent self-organization process had been fully restored (Supplemental Figure S7B). To determine whether each individual feather converted into an aster once dynein became active we rapidly squashed the extract after dilution between passivated microscopy glasses and followed the MT feathers by live imaging. We indeed observed that all the individual feathers converted into asters within around 5 min (Figure 6B; Supplemental Movie 8). A closer examination showed a gradual change in MT organization as tubulin redistributed and accumulated at the position of the feather vertex while the angular distribution of MTs increased over time (Supplemental Figure S7C).

**Figure:** Movie S8 **RanGTP feathers release from dynein inhibition** Live confocal fluorescence microscopy of RanGTP feathers released from dynein inhibition. Video captions are shown in Fig. 2A and 2B. Samples were squashed between PLL‐passivated glass. MTs were visualized through Atto647‐tubulin fluorescence. Images were collected every 2 s. Time frame 15 fps. Scale bar, 50 μm.

**FIGURE 7:**
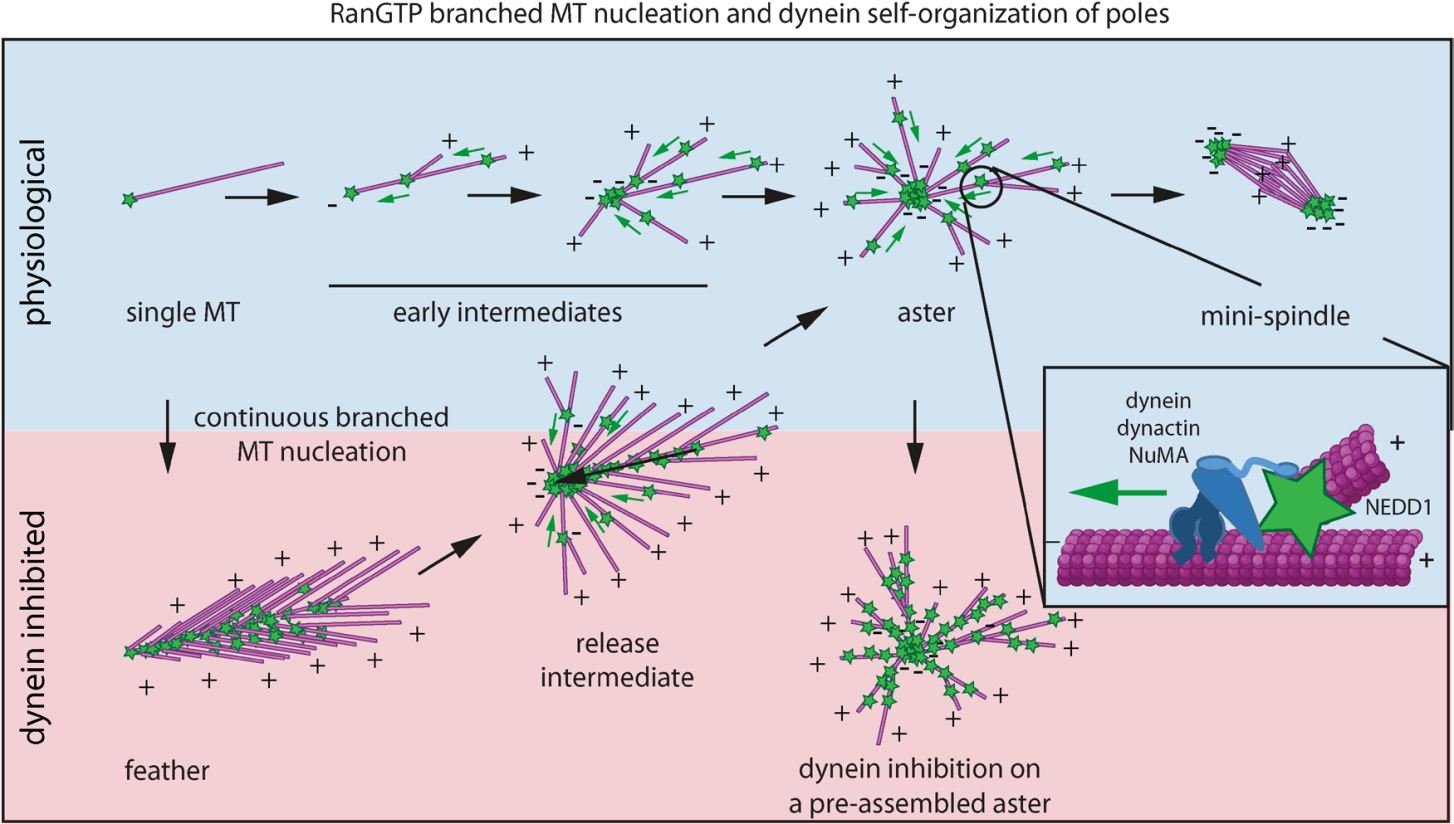
Model of RanGTP aster pole self-organization. Under physiological conditions (top, blue shade), dynein-mediated transport and MT branching act in synergy to assemble the pole of an aster. Under dynein inhibition (bottom, red shade), NEDD1 accumulates all along the MTs while continuous nucleation drives the assembly of static branched structures (feathers). Dynein inhibition can be reverted and feather branched MTs then reorganize into asters reestablishing the physiological RanGTP MT self-organization process. Inhibition of dynein on preassembled asters does not affect astral MT organization and blocks NEDD1 and branched MTs transport. MTs are shown in pink. MT plus and minus ends are indicated by “+” and “–”, respectively. Green stars indicate NEDD1 (visualized through the fluorescently labeled anti-NEDD1 antibody in our experiments) and the complex formed by dynein, dynactin and NuMA is in blue. Green arrows indicate the direction of motion and black arrows the temporal sequence of events.

These data demonstrate that dynein can directly organize the branched MTs of a feather suggesting a model where, under normal conditions, dynein-dependent transport and branched MT nucleation cooperate to organize asters with focused poles (Figure 7).

## DISCUSSION

Our study addressed the apparent conflict between two concepts that are essential for spindle assembly: dynamic dynein-dependent transport of MTs along other MTs (Merdes *et al.*, 1996, 2000; Karsenti and Vernos, 2001) and branched MT nucleation generating polarized MT arrays (Goshima *et al.*, 2008; Petry *et al.*, 2011; Petry *et al.*, 2013). Studying aster formation in *Xenopus* egg extract, we found that the branched MT network architecture is only static when dynein is inhibited, and that MTs, together with MT nucleators, redistribute under normal conditions toward poles in a dynein-dependent manner. Dynein-dependent organization of nucleators has also been observed in other systems and seems to be a general mechanism employed in different cellular contexts to organize dynamic MTs into poles (Vorobjev *et al.*, 2001; Malikov *et al.*, 2004).

We found that the classical RanGTP MT feathers that form when dynein is inhibited (Petry *et al.*, 2013) reorganize into asters after releasing them from dynein inhibition. Under normal conditions, NEDD1-labeled branch points and MTs move together with similar velocities toward the poles, consistent with a flux of MT minus ends previously measured in mitotic spindles in human cells (Lecland and Luders, 2016).

Our results highlight that MT branching and dynein transport cooperate during RanGTP MT aster self-organization. This is different from Taxol and DMSO aster formation where dynein crosslinks and transports MTs that nucleate randomly in the cytoplasm in a RanGTP-independent manner (Verde *et al.*, 1991; Wilde and Zheng, 1999). Although we were not able to visualize directly the transport of individual branched MTs inside an aster by fluorescence microscopy due to the high MT density in asters, our computer simulations lent support to the interpretation that dynein/dynactin/NuMA may directly pull on the NEDD1/nucleators, therefore, sliding branched MT minus ends toward other MT minus ends. Such a mechanism is sufficient to reproduce in simulations the assembly of an aster with a focused pole and the reorganization of a RanGTP MT feather into an aster when dynein becomes active, in good agreement with our experimental observations.

Our work reconciles two seemingly incompatible principles of MT self-organization and suggests potential additional benefits of branched MT nucleation and dynein-dependent transport working together to ensure correct spindle pole self-organization. First, local MT amplification by branched nucleation produces a MT network with connections that dynein can readily work on. Second, dynein does not only focus MT minus ends, as previously thought, but also nucleation factors, reinforcing pole stability. Understanding if and how dynein directly pulls and slides the large complex of proteins constituting MT branch points will be an important goal of future studies.

## MATERIALS AND METHODS

Request a protocol through *Bio-protocol*.

### *X. laevis* egg extract

All experiments were performed in the *X. laevis* egg extract system. All work involving animals was done according to standard protocols approved by the Centre for Genomic Regulation Ethics Committee. Cytostatic factor–arrested egg extracts from *X. laevis* were prepared from adult females of 125 g weight of average. Animals were purchased from Xénopes (http://xenopus.univ-rennes1.fr) and from Xenopus1 Corp. (www.xenopus1.com).

Frogs were primed with 100 UI of pregnant mare serum gonadotropin (581134.5, MSD) at least 4 d before human chorionic gonadotropin injection (C1063, Sigma). Extracts were prepared as previously described (Desai *et al.*, 1999; Peset *et al.*, 2005) with the following specifications: 1) Eggs were initially packed by a centrifugation at 200 × *g* for 1 min. Excess of buffer was removed and the eggs were covered by silicone oil AP 100 (10838, Sigma). A second centrifugation at 400 × *g* for 1 min fully packed the eggs. Oil and buffer were finally removed. 2) Eggs were crushed by centrifuging 20 min at 17000 × *g* at 18°C. Extracts were tested for RanGTP MT aster assembly and used fresh or after snap freezing in liquid nitrogen.

### Antibodies

The rabbit anti *Xenopus* NEDD1 was raised against the C-terminal of the protein (371-655). Antibody specificity was shown by depletion and add-back experiments as shown in previous works (Pinyol *et al.*, 2013; Scrofani *et al.*, 2015). NHS-Atto647 (07376, Sigma) or NHS-Atto488 (41698, Sigma) were coupled to the anti-NEDD1 antibody using on-resin IgG labeling as previously described (Field *et al.*, 2017). The anti-NuMA inhibitory serum was prepared against the distal tail as described previously (Merdes *et al.*, 1996). IgG were purified from 1 ml of the serum using Protein G Sepharose (17-0618-01, GE Healthcare) loaded on 100 μl tips homemade columns as previously described (Field *et al.*, 2017).

### Protein expression and purification

The His-RanQ69L mutant (RanGTP) was expressed in *Escherichia coli* BL21 DE3 strain and affinity purified using Ni-NTA resin (1018244, Qiagen). Fractions were pulled and buffer was changed to CSF-XB buffer (10 mM HEPES [pH7.7], 100 mM KCl, 0.1 mM CaCl_2_, 2 mM MgCl_2_, 5 mM EGTA, and sucrose 50 mM) using PD-10 desalting columns (17-0851-01, GE Healthcare). After quantification the protein was diluted to 4 mg/ml and supplemented while gently steering with 1 mM GTP and 20 mM MgCl_2_. The protein was then concentrated at 220 μM. The recombinant GFP-AuroraA was expressed in *E. coli* BL21(DE3)-pLysS and purified as previously described (Sardon *et al.*, 2008). The recombinant GFP-TPX2 was expressed and purified as previously described (Brunet *et al.*, 2004). The p50/Dynamitin and p150-CC1 were expressed from *E. coli* BL21 RIL and purified as described previously (Wittmann and Hyman, 1999; Uteng *et al.*, 2008). Bovine brain tubulin was prepared as described (Hyman *et al.*, 1991) and coupled with NHS-Atto488 (41698, Sigma), NHS-Atto647 (07376, Sigma), or TAMRA (C1171, Invitrogen).

### MT assembly reactions

RanGTP, Paclitaxel (T7402, Sigma), and DMSO (D4540, Sigma) MT assembly reactions were prepared on ice adding 11 μM RanGTP, 0.1 μM Paclitaxel, or 0.5% DMSO, respectively. Sodium orthovanadate (vanadate; 450243, Sigma) was used at 0.5 mM for RanGTP feather assembly and at 2.5 μM for the release from dynein inhibition experiments. Recombinant p50 was used at 1 mg/ml. 250–500 nM of fluorescently labeled tubulin was added to visualize MTs. The polyclonal anti-NEDD1 antibody was added at 0.5–1 μM. Reactions were incubated at 20°C or processed for live imaging at room temperature. When specified, RanGTP aster and feathers were preassembled for 15 min at 20°C and then studied under different experimental conditions. For dynein inhibition on preassembled structures, vanadate was added at 0.5 mM. For the release of feathers from dynein inhibition samples were fixed immediately before the release to check for feather shape. Feathers with comparable shape were used for the experiments.

### Microscopy

For fixed samples imaging, 1 μl of extract was added to 5 μl of 11% formaldehyde, 48% glycerol in Marc’s modified Ringer’s buffer (5 mM HEPES, 100 mM NaCl, 2 mM KCl, 1 mM MgCl_2_, 0.1 mM EDTA, and pH 7.8) and squashed under an 18 × 18 mm glass coverslip. In live epifluorescence and confocal imaging experiments, 6 μl of egg extract were squashed under an 18 × 18 mm coverslip passivated with PLL-g-PEG (20)-[3.5]-(2; SZ42-90, SuSoS) resulting in a sample thickness of about 20 μm. For TIRF microscopy live imaging, 10 μl of egg extract were flushed into a flow chamber assembled with a coverslip and a slide held together with two stripes of double-sided tape (05338, Tesa). For all live imaging experiments, samples were sealed with Korasilon paste (Kurt Obermeier GmbH & Co.).

Wide-field epifluorescence microscopy was performed using a 40 × or a 100 × objective on an inverted DMI-6000 Leica microscope with a sCMOS camera (DFC9000GT, Leica) and a SOLA light engine (Lumencor). On live acquisitions of NEDD1 speckles, images were taken every 0.5 s. On live acquisitions of NEDD1 and tubulin speckles (two channel), images were taken every 3 s. Confocal imaging of fixed samples was made using a 63 × objective on a TCS SP5 microscope (Leica). Z-stacks were acquired covering the whole aster/feather volume. Live confocal fluorescence microscopy was performed on a custom assembled system (Cairn Research) built around an Eclipse T*i* (Nikon) inverted microscope using a X-Light V2 (CrestOptics) spinning disk unit, a 40 × oil-immersion objective (CFI60 Plan Fluor NA 1.3, Nikon), and a sCMOS camera (Prime95B, Teledyne Photometrics). For RanGTP aster and feather assembly experiments, Z-stacks of images were taken every 15 s. For the release from dynein inhibition experiments, Z-stacks of images were taken every 2 s. Live TIRF microscopy imaging was done using a custom assembled system (Cairn Research) built around an Eclipse T*i* (Nikon) inverted microscope using an azimuthal TIRM unit (iLas2, Gataca Systems), a 100 × oil-immersion objective (CFI SR Apo TIRF NA 1.49, Nikon), and an EMCCD camera (iXon 888 Ultra, Andor). Images were acquired every 0.5 s. All images have been processed using ImageJ (Wayne Rasband, http://imagej.nih.gov/ij).

### Quantification and statistical analysis

To quantify radial intensity profiles on RanGTP asters and feathers, a segmented line selections were manually defined from the asters poles and feathers vertexes to the periphery. Tubulin and NEDD1 intensity profiles were then calculated and corrected for the background. The background was estimated by the average intensity from a line selection placed outside the structure.

To quantify tubulin intensity around the centers of aster or at the vertexes of feathers circular selections were manually defined. Signal intensities were calculated and corrected for the background. The background was calculated from three line ROI placed randomly outside each structure. Because the high variability among independent experiments we: 1) calculated average intensities for each experiment, 2) calculated the fold difference of feathers versus asters, 3) averaged the fold differences and plotted them with SD. Graph Pad software (www.graphpad.com) for statistical significance.

For NEDD1 speckle count segmented line selections were placed over bundles of MTs from asters poles and feathers vertexes to the periphery. Background was subtracted as explained before. The number of NEDD1 speckles estimated counting manually the number of peaks inside 20-μm long segment defined starting 5 μm away from the pole/vertex.

NEDD1 molecules were automatically tracked with FIESTA (Ruhnow *et al.*, 2011). Tracks were manually verified to remove crossing tracks or falsely linked tracks (due to high density of NEDD1 molecules). The NEDD1 signal was blurred using a Gaussian filter (σ = 10 pixel) and the center of each blurred aster was used for drift correction. For feathers, a nonmoving NEDD1 molecule was used for drift correction. Drift correction was applied after tracking, so only unmodified images were used for tracking of the individual NEDD1 molecules. Due to the high signal of the aster center/feather tip tracking failed near the center/tip due to a nonhomogenous background when fitting the two-dimensional Gaussian.

For NEDD1 molecules and MT speckles analysis the tracking approach was modified and combined with PIV. Briefly: 1) Images with less than 100-nm pixel size were downscaled using 2 × 2 binning average. 2) Original (or binned) images were blurred using a Gaussian filter (σ = 10 pixel) and the center of the blurred aster was tracked for drift correction. 3) Original (or binned) images were subjected to a background correction using a top-hat filtering disk with radius *r* = 5 pixel (rolling ball correction). Individual particles were tracked in the first 10 s of each time-lapse (using a relative threshold of the 1.4*median image intensity – in FIESTA relative threshold = 140%). 4) Particles with an amplitude less than 2*σ of the background noise were disregarded. 5) Particle positions were drift corrected and each position was matched to the closest position in the following frame (if possible). 6) The distance to the aster center for each position was calculated and used to estimate the velocity and corresponding distance from the aster center. 7) Matched positions exceeding a maximum distance D_max_ = 500 nm and intensity changes of more than 20% were excluded from the PTV analysis. NEDD1 molecules and MT speckles were analyzed with the same algorithm.

### Computer simulations

We used Cytosim (Francois Nedelec, 2007) to simulate the interplay between branched MT nucleation and the dynein motor activity. Our model includes three key components: MTs, branching nucleators, and dynein/NuMA-like motor complexes.

MTs were represented as flexible filaments, exhibiting diffusion and steric interactions. MT minus ends were static and for MT plus ends we adopted a two-state model for dynamic instability, with parameters resembling those found in wild-type egg extracts (growth speed: 0.18 µm/s; catastrophe frequency: 0.03/s; rescue frequency: 0.013/s; shrinkage speed: –0.58 µm/s; Tournebize *et al.*, 2000). The mean length of the MT was established at 7 µm.

Nucleators were explicitly modeled. Upon irreversibly binding to a MT, a nucleator initiates the formation of a new MT at an angle of 5 degrees. This angle is constrained by a spring-like force, characterized by a stiffness parameter. The nucleator can also diffuse along the mother MT and slide in response to applied force. The speed of the nucleator’s movement is dependent on the inverse of its diffusion coefficient and the applied force. This diffusion coefficient was set at 0.000126 µm^2^/s to hinder dynein-mediated transport as observed in experiments.

The dynein/NuMA motor was modeled as an asymmetric entity, comprising a MT minus end binding unit (mimicking the function of NuMA in complex with dynein [Hueschen *et al.*, 2017]) and a minus end directed motor unit. These two units were interconnected through a Hookean link, with the end binding unit remaining static and the motor unit capable of unidirectional movement. The unloaded motor velocity was 0.8 µm/s and speed decreased linearly with force.

All simulations were conducted within a thin flat, three-dimensional space measuring 70 × 70 × 0.2 µm, featuring reflecting boundaries in all dimensions. The viscosity of the medium was set at 0.03 pN s/µm^2^ to match that of *Xenopus* egg extract. We used 270 nucleators and 5400 dynein motors in the simulation. Other parameters are as described previously (Chew *et al.*, 2023).

For tubulin speckle and nucleator speed estimates, we chose random points along the MT as “tubulin speckles.” The instantaneous speeds of tubulin speckles and nucleators during aster formation were calculated from snapshots taken at intervals of 4.5 s over a 10-s time window.

## Supplementary Material



## Abbreviations used:

DMSO: dimethyl sulfoxide
GTP: guanosine triphosphate
MT: microtubule
PIV: particle image velocimetry
SD: standard deviation
*X. laevis*: *Xenopus laevis*
