# Supplementary material for: Branched microtubule nucleation and dynein transport organize RanGTP asters in Xenopus laevis egg extract
Source: Mol Biol Cell. 2023 Nov 22;35(1):ar12. doi: 10.1091/mbc.E23-10-0407 (PMC10881172; doi:10.1091/mbc.E23-10-0407)
Supplement: Supplementary file 9 [file mbc-35-ar12-s001.pdf]

Supplemental Materials

Molecular Biology of the Cell

Scrofani *et al*.

Supplemental material

Branched microtubule nucleation and dynein transport organize RanGTP asters in *Xenopus laevis* egg extract

Jacopo Scrofani[1*], Felix Ruhnow[1], Wei-Xiang Chew[1], Davide Normanno[1,#], Francois Nedelec[2], Thomas Surrey[1,3,4] and Isabelle Vernos[1,3,4,*]

[1] Centre for Genomic Regulation (CRG), The Barcelona Institute of Science and Technology, Barcelona, Spain

[2] Sainsbury Laboratory, Cambridge University, Bateman street, CB2 1LR Cambridge, UK

[3] Universitat Pompeu Fabra (UPF), Barcelona, Spain

[4] ICREA, Pg. Lluis Companys 23, Barcelona 08010, Spain

[#] present address:

Institute of Human Genetics (IGH), Univ Montpellier, CNRS, Montpellier, France

* correspondence:

isabelle.vernos@crg.eu

jacopo.scrofani@crg.eu

Supplemental figures

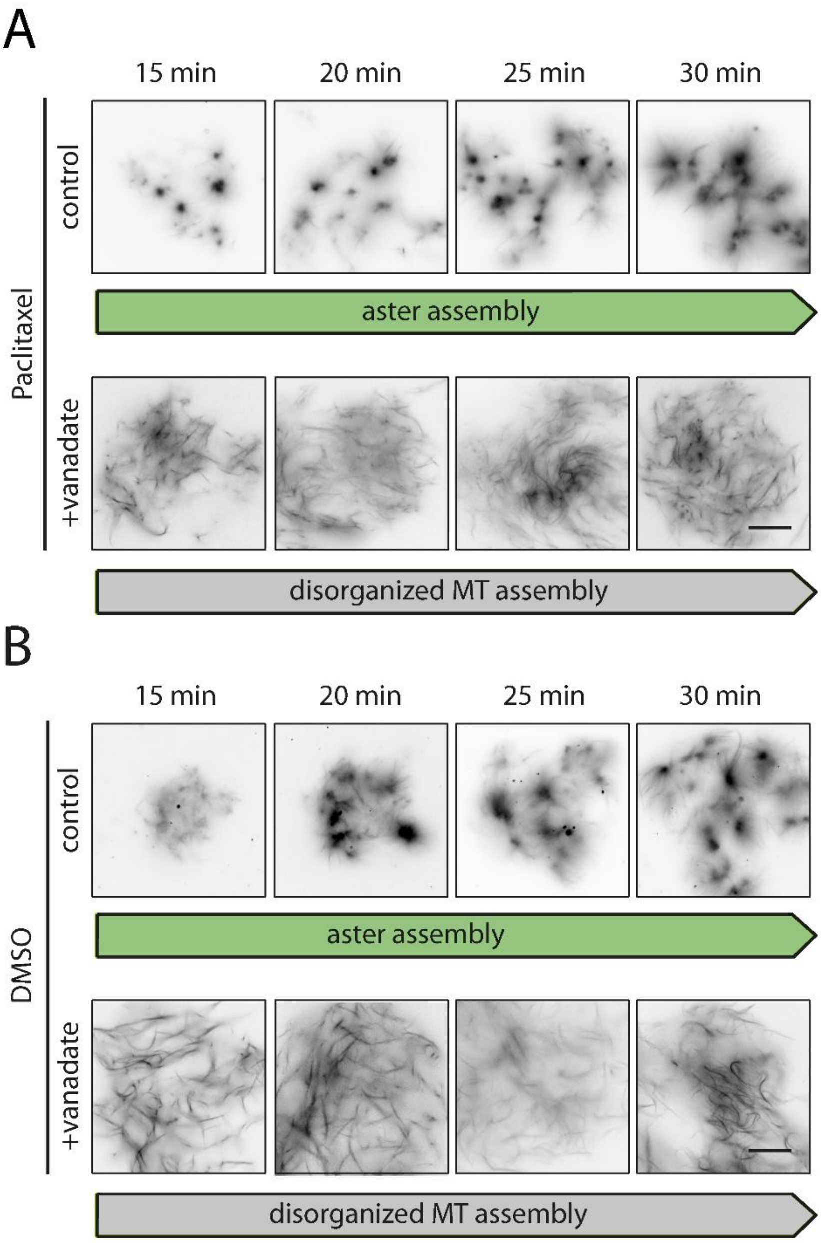

Figure S1: A) MT structures formed in egg extract incubated with Paclitaxel or B) with DMSO with (+vanadate) or without (control) vanadate. Samples were collected and fixed at the indicated time points.

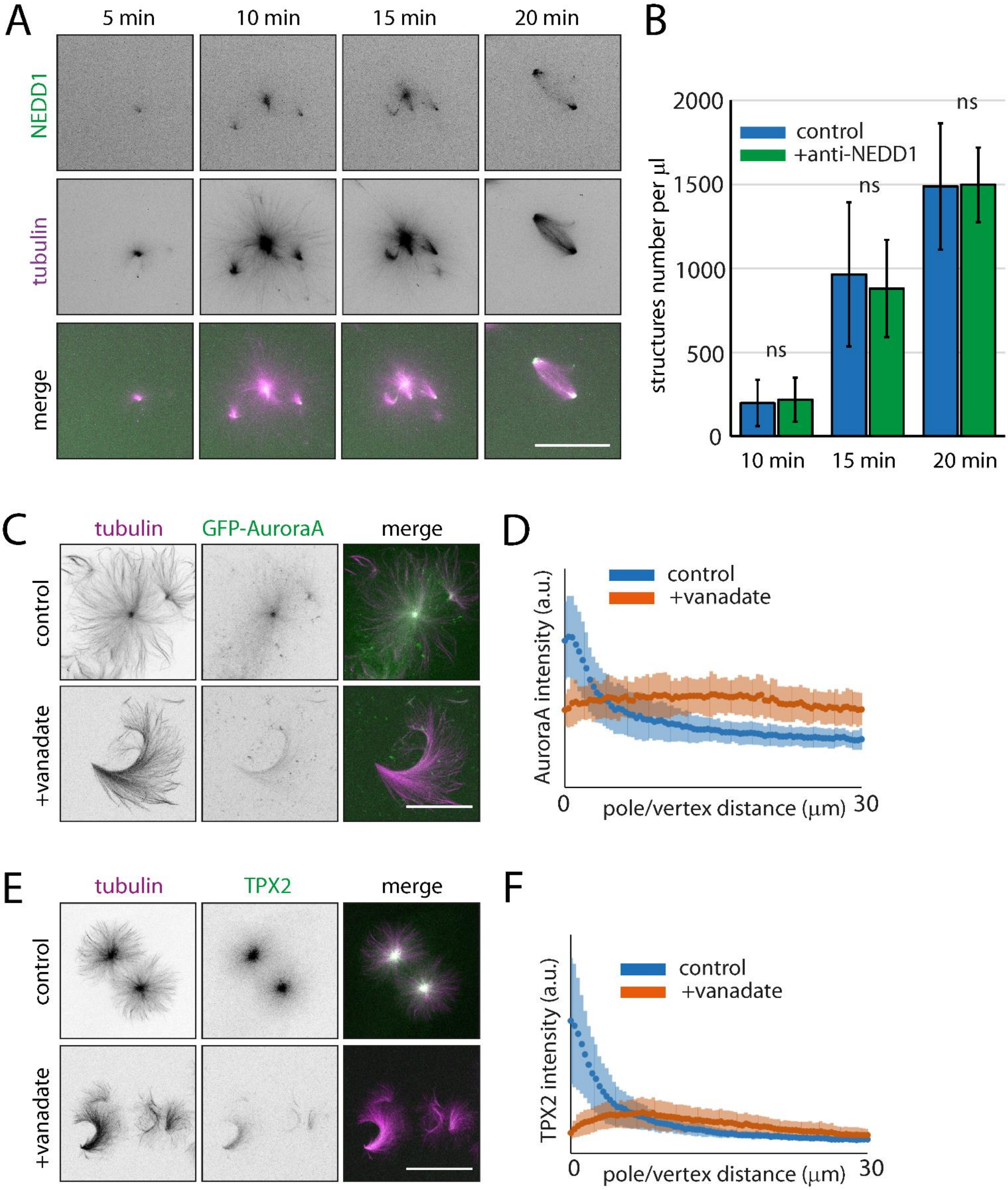

Figure S2: A) Representative images from a live confocal movie of RanGTP aster assembly. MTs were visualized through Atto647-tubulin fluorescence (magenta in composite images) and NEDD1 via anti-NEDD1-Atto488 antibody (green in composite images). Scale bar, 50 µm. B) Means of the number of RanGTP asters counted in control extracts or extract in the presence of the anti-NEDD1 antibody and at different times as indicated. Four independent experiments were performed. Error

bars are standard deviations. ns is not significant (Student t-test). C-F) GFP-Aurora A and GFP-TPX2 localizations on RanGTP asters and feathers. Images are maximum projections from confocal images of squashes prepared from fixed samples. Graphs are intensity profiles of GFP-Aurora A and GFP-TPX2 on asters (control) and feathers (+vanadate). The profiles were obtained from selected MTs along the pole/vertex to periphery axis. Data points correspond to averages and shaded areas to standard deviation from 50 profiles from one representative experiment.

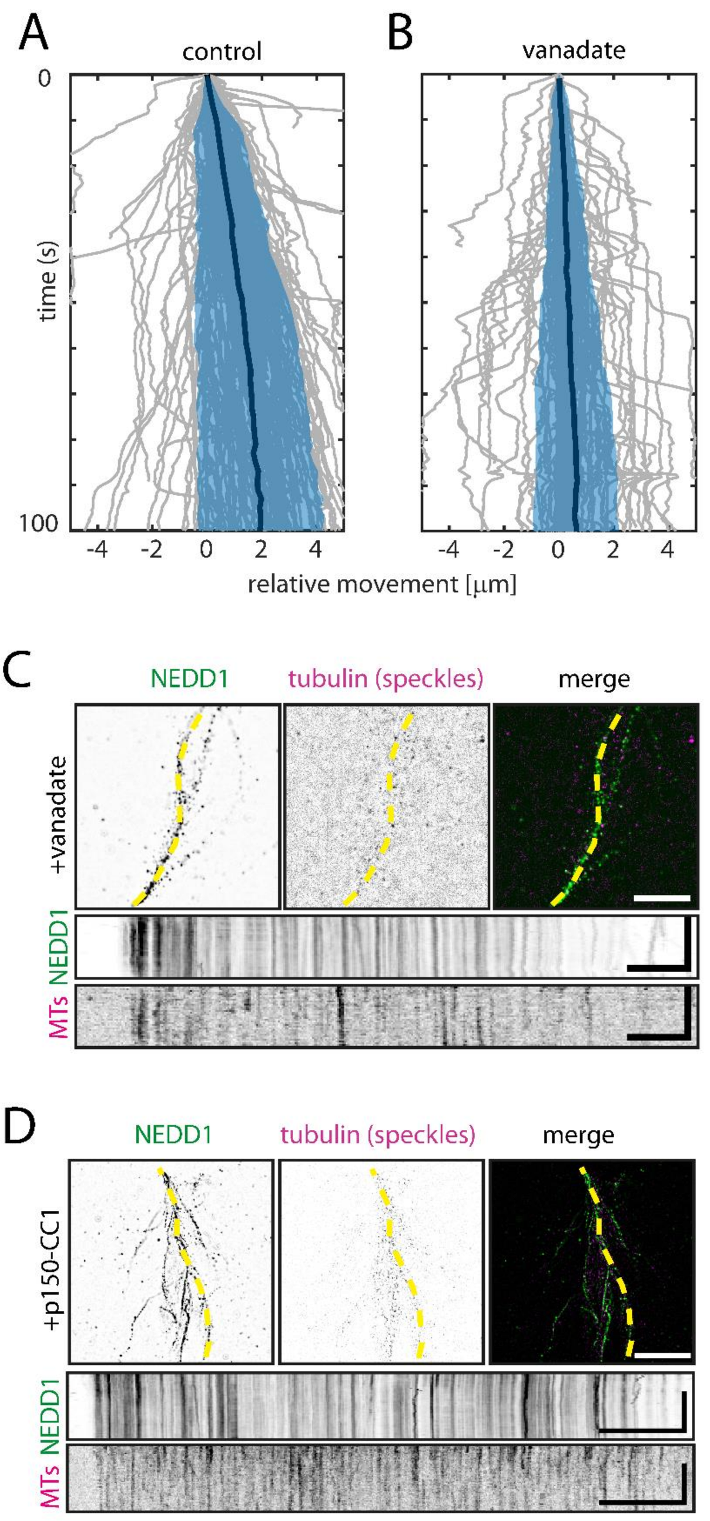

Figure S3. A, B) Individual NEDD1 tracks analysis. Each track represents the relative movement of a NEDD1 speckle towards the pole/vertex (positive values) or towards the periphery (negative values). Dark blue line is the average track and shaded area is the standard deviation. n=217 (asters) and n=192 (feathers) tracks from three asters and three feathers imaged in three independent experiments.. C, D) Live epifluorescence microscopy of NEDD1 and MT speckles on RanGTP feathers assembled with vanadate or p150-CC1 as indicated (top). Representative

kymographs of NEDD1 and MTs speckles prepared on the indicated selection (yellow dashed lines) (bottom). Scale bars, 20μm (images), x=120s, y=10μm (kymographs).

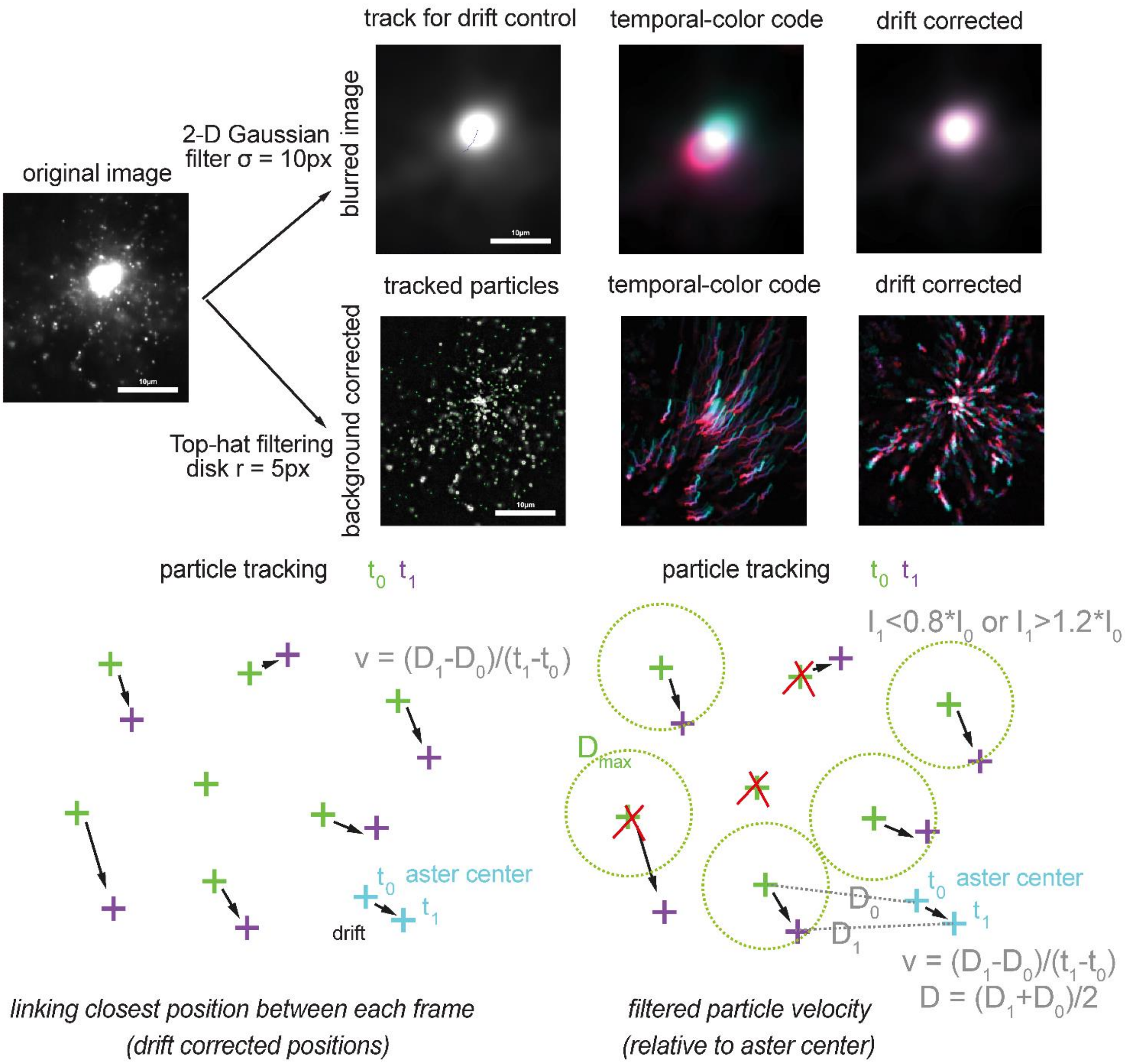

Figure S4

Experimental workflow for Particle Image Velocimetry (PIV) on NEDD1 and MT speckles on RanGTP asters.

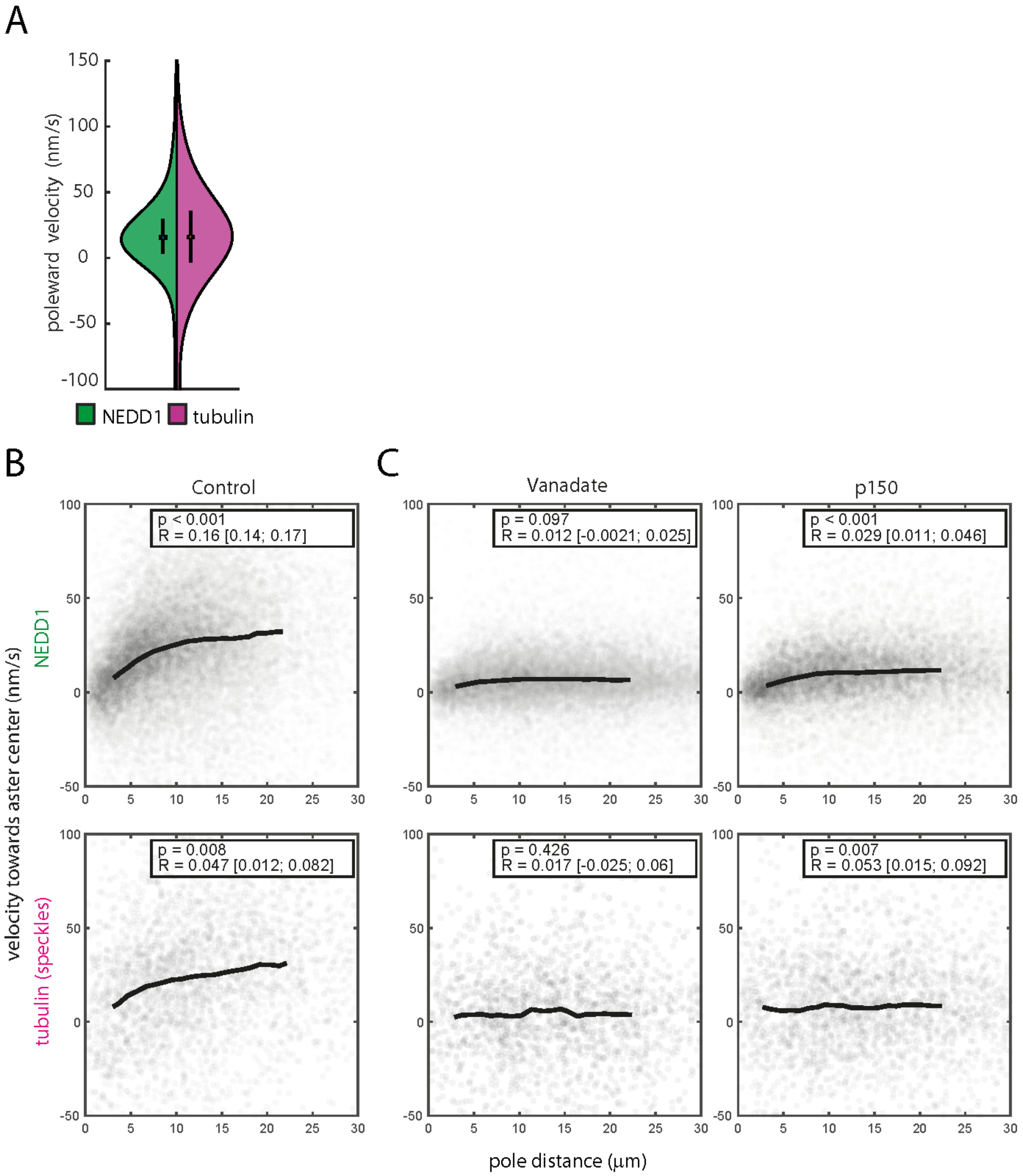

Figure S5. A) Speckle velocity measured by particle image velocimetry on one representative aster (same as Fig. 3F). Quantifications correspond to 120 seconds. Half violin plot represent probability density for NEDD1 and MTs speckles velocities (NEDD1: n=9593; tubulin: n=4096). Error bars are the median and interquartile range. p: Mann-Whitney U-test. B, C) NEDD1 and MT speckle velocity correlation with the distance from the aster pole in control, vanadate or p150-CC1 –treated extracts. Quantifications correspond to 10 seconds. Black lines are the median velocities from all speckles

measured at a certain distance from the pole. R is Pearson's coefficient for velocity/distance correlation with confidence intervals (in brackets). P-value is correlation significance. In controls (NEDD1: N=45 asters and n=18434 speckles; tubulin: N=26 asters and n=3152 speckles), in +vanadate (NEDD1: N=24 asters and n=20453 speckles; tubulin: N=16 asters and n=2129 speckles), in +p150-CC1 (NEDD1: N=17 asters and n=12209 speckles; tubulin: N=17 asters and 2575 speckles) from three independent experiments.

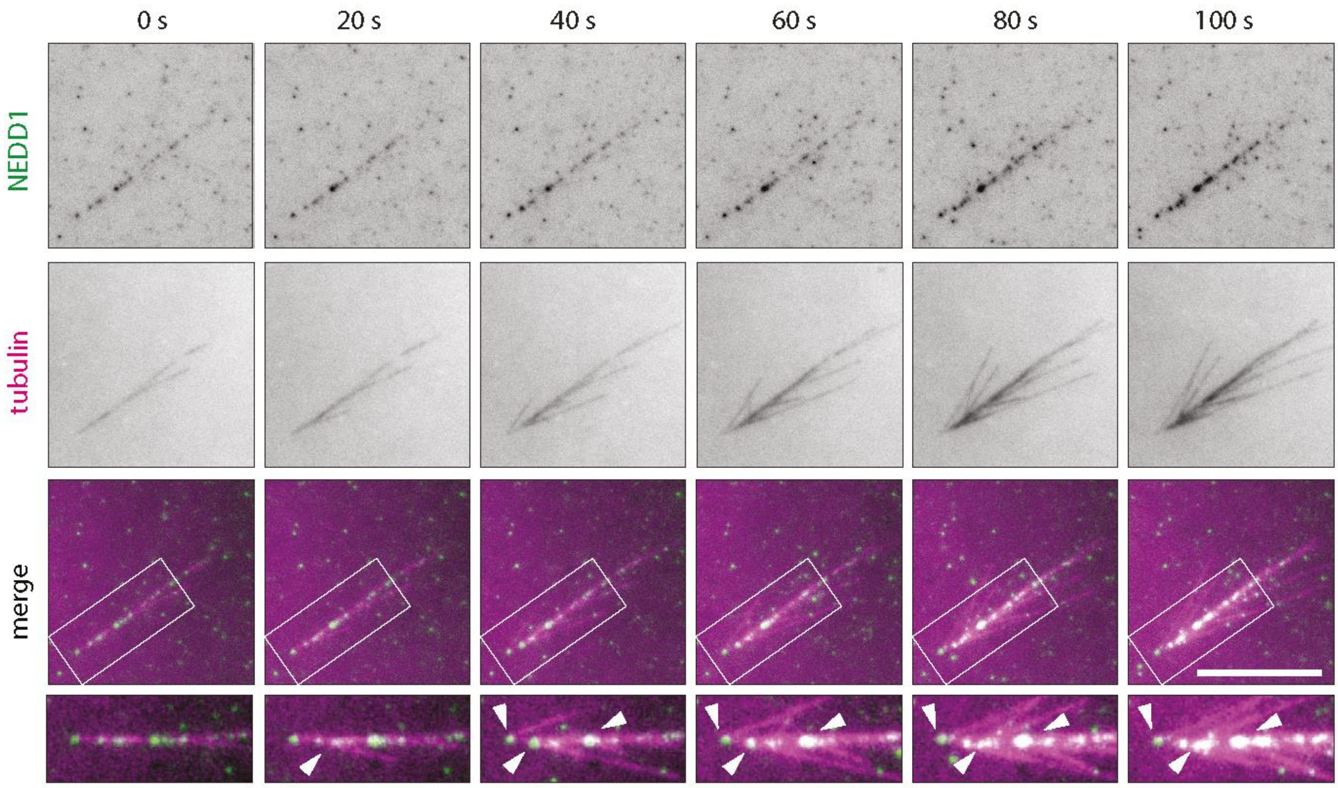

Figure S6

Representative images from a live TIRF movie of RanGTP feathers (Movie 4, right). White rectangles indicate the magnified areas shown at the bottom. Arrows indicate some of the co-localization between NEDD1 speckles and branched MTs. MTs were visualized through Atto647-tubulin fluorescence (magenta in composite images) and NEDD1 via anti-NEDD1-Atto488 antibody (green in composite images). Scale bar, 20 μm.

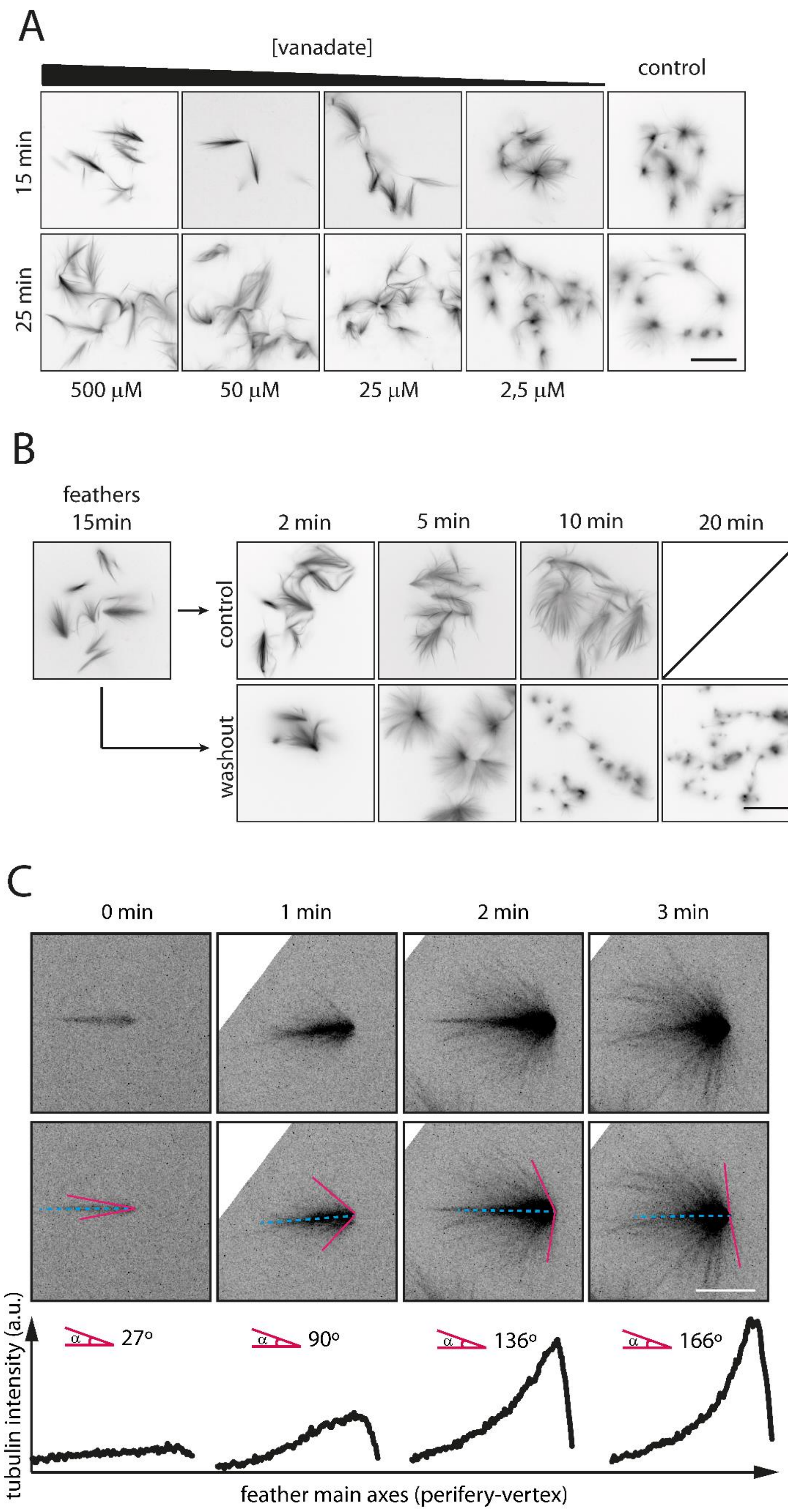

Figure S7. A) RanGTP MT assembly in the presence of different vanadate concentrations. Images are representative squashes from fixed samples prepared at the indicated time points. The control

shows RanGTP asters assembled without vanadate. MTs were visualized through rhodamine-tubulin fluorescence. Scale bar, 50 μm. B) Representative squashes from fixed samples of RanGTP feathers assembled with vanadate and fixed at the indicated time (top). Feathers were released from dynein inhibition (bottom). MTs were visualized through rhodamine-tubulin fluorescence. Scale bar, 50 μm. C) Detailed view of the conversion of a RanGTP feather into aster that is associated with changes in MT angular distribution (top panel). MTs were visualized through Atto488-tubulin fluorescence. Scale bar, 10 μm. Tubulin intensity profiles (bottom panel) were measured along the main feather axis (middle panel, blue dashed lines). Angular values (α, indicated by red lines in the middle panel) indicate the angle of the branched-MT distribution over time after release.

Supplemental movies

Movie 1. RanGTP asters and feathers assembly

Live confocal fluorescence microscopy of RanGTP aster (left) and feather (right) assembly. Samples were squashed between PLL-passivated glass. MTs were visualized through Atto647-tubulin fluorescence. Images were collected every 15 s. Time frame 15 fps. Scale bar, 50 μm.

Movie 2. NEDD1 speckles on RanGTP asters and feathers

Live epifluorescence microscopy of RanGTP asters and feathers. Speckles were tracked automatically and their velocity toward the aster center or feather vertex (blue dot) measured. Inward slow (green) and fast (light blue) and outward (magenta) tracks were visualized. NEDD1 was visualized via anti-NEDD1-Atto488 antibody staining of the endogenous protein (right). Images were collected every 0.5 s. Time frame 15 fps. Scale bar, 10 μm.

Movie 3. NEDD1 and tubulin speckles on RanGTP asters and feathers

Live epifluorescence microscopy of RanGTP asters and feathers. NEDD1 was visualized via anti-NEDD1-Atto488 antibody staining of the endogenous protein (right). Tubulin speckles were

visualized by a low concentration of rhodamine tubulin. Images were collected every 2 s. Time frame 15 fps. Scale bar, 10 μm.

Movie 4. NEDD1 localization on RanGTP branched MTs minus ends

TIRF microscopy of RanGTP feathers. Video captions are shown in Fig. S6C. NEDD1 was visualized via anti-NEDD1-Atto488 antibody staining of the endogenous protein (right). MTs were visualized by Atto647 tubulin. Images were collected every 2 s. Time frame 15 fps. Scale bar, 10 μm.

Movie 5. Computer simulation of branched MT nucleation without dynein

MTs (gray) and nucleators (red).

Movie 6. Computer simulation of branched MT nucleation with dynein

MTs (gray), nucleators (red) and dynein (blue).

Movie 7. Computer simulation of feather MT organization after dynein addition

MTs (gray), nucleators (red) and dynein (blue).

Movie 8. RanGTP feathers release from dynein inhibition

Live confocal fluorescence microscopy of RanGTP feathers released from dynein inhibition. Video captions are shown in Fig. 2A and 2B. Samples were squashed between PLL-passivated glass. MTs were visualized through Atto647-tubulin fluorescence. Images were collected every 2 s. Time frame 15 fps. Scale bar, 50 μm.
